# Supplementary material for: Effect of Water-Soluble Polymers on the Rheology and Microstructure of Polymer-Modified Geopolymer Glass-Ceramics
Source: Materials (Basel). 2024 Jun 11;17(12):2856. doi: 10.3390/ma17122856 (PMC11204717; doi:10.3390/ma17122856)
Supplement: Supplementary file 1 [file materials-17-02856-s001.zip › materials-3024306-supplementary.pdf]

**Supporting Information**

# Effect of water-soluble polymers on the rheology and microstructure of polymer-modified geopolymer glass-ceramics

John M. Migliore[1,2,3], Patrick Hewitt[2,3], Theo J. Dingemans[1], Davide L. Simone[2], William Jacob Monzel[2,*]

[1] Department of Applied Physical Sciences, The University of North Carolina at Chapel Hill, Chapel Hill, NC 27599, United States

[2] Materials and Manufacturing Directorate, Air Force Research Laboratory, AFRL/RXNP, Dayton, OH 45324, United States

[3] UES, Inc. A BlueHalo Company, Dayton, OH 45432, United States

*Corresponding author email: william.monzel.1@us.af.mil

**Table S1**. Chemical stability of PBDT and PBDI.

| Solution* | Heating Conditions | Solution Appearance |
|---|---|---|
| PBDT in 13M phosphoric acid | 25 ºC, 4 weeks | Undissolved |
| PBDT in 9M phosphoric acid | 25 ºC, 1 week | Undissolved |
| PBDT in 9M phosphoric acid | 25 ºC, 4 weeks | Clear-translucent, Viscous |
| PBDT in 9M phosphoric acid | 55 ºC, 8 h | Clear-translucent, Viscous |
| PBDT in 9M phosphoric acid | 55 ºC, 16 h / 85 ºC, 3 h | Clear-colorless, Viscous** |
| PBDI in 9M phosphoric acid | 25 ºC, 24 h | Clear-translucent, Viscous |
| PBDI in 9M phosphoric acid | 55 ºC, 8 h | Clear-colorless, Viscous** |

*All solutions contained 1.0 wt. % polymer. The solutions were sealed in 25 mL Pyrex® media bottles under $N_2$ atmosphere and lightly stirred.

**After heating at 85 ºC, solution flowed readily compared to the gel-like behavior of the PBDT 9M phosphoric solutions at 55 ºC and below. Flow behavior was similar to PBDI in 9M phosphoric acid at 55 ºC.

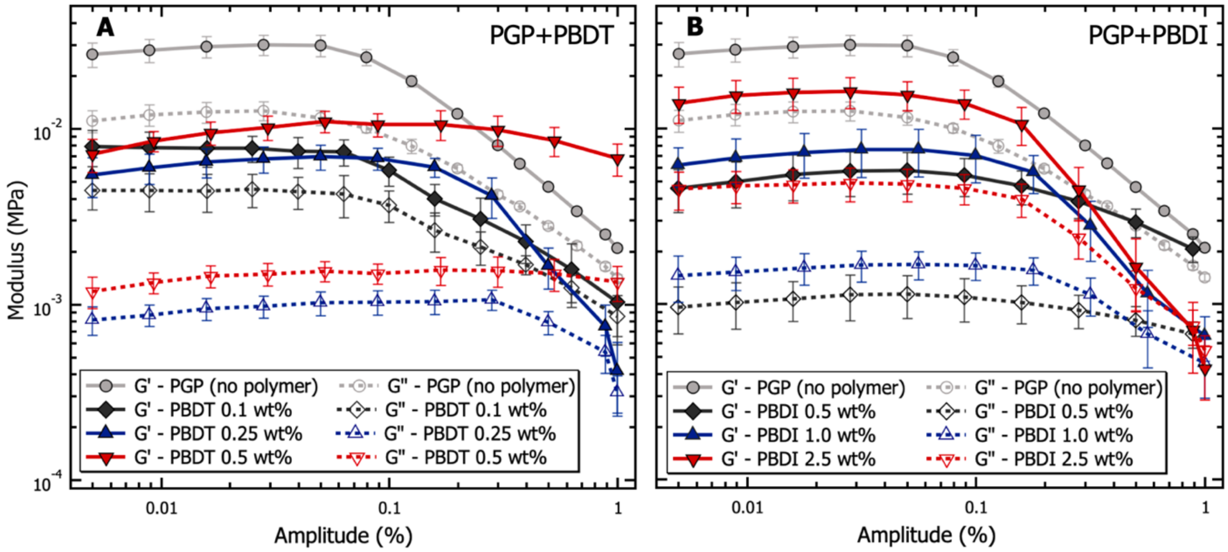


**Figure S1**. Analysis of the linear viscoelastic regime as a function of amplitude (percent strain) for PGP resins containing (**A**) 0.1, 0.25, 0.5 wt. % PBDT and (**B**) 0.5, 1.0, 2.5 wt. % PBDI. Samples utilized 25 mm stainless steel parallel plates, 1 Hz frequency, 25 ºC temperature control, and all experiments were performed in triplicate.

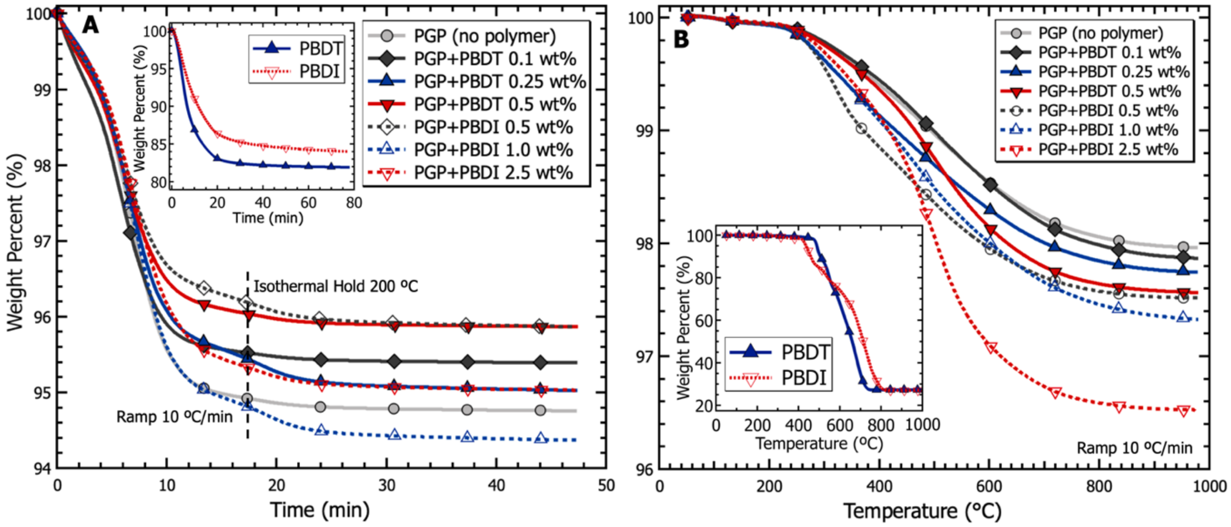


**Figure S2**. (**A**) Quantification of water uptake for PGP composite materials. Samples were heated to 200 ºC at 10 ºC/min and held at 200 ºC for 30 min. (**Inset A**) Water uptake of PBDT and PBDI. The polymers were heated to 200 ºC at 10 ºC/min and held at 200 ºC for 1 h. (**B**) Thermal gravimetric analysis of PGP composites. All samples were dried at 200 ºC for 30 min prior to final heat ramp of 10 ºC/min up to 1000 ºC in air. (**Inset B**) Thermal degradation of PBDT and PBDI at 10 ºC/min up to 1000 ºC in air.

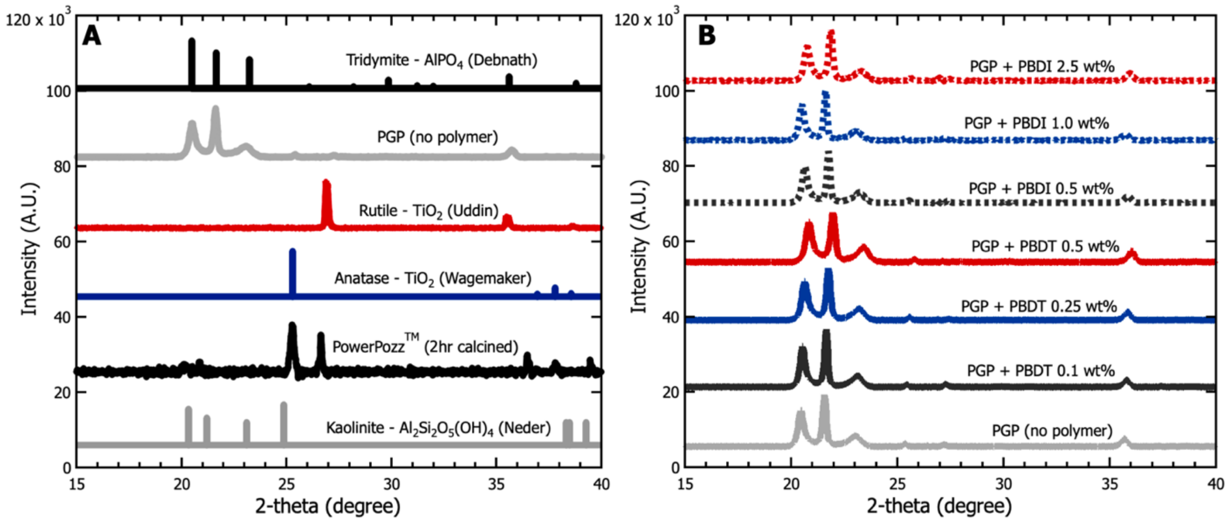


**Figure S3**. (**A**) Comparison of phosphate geopolymer diffraction pattern to other $TiO_2$, $AlPO_4$, and $SiO_2$ sources [1–5]. (**B**) X-ray powder diffraction of PGP and PGP + Polymer composites. Diffraction peaks emerge between 20º–24º and 35º–36º (2-theta).

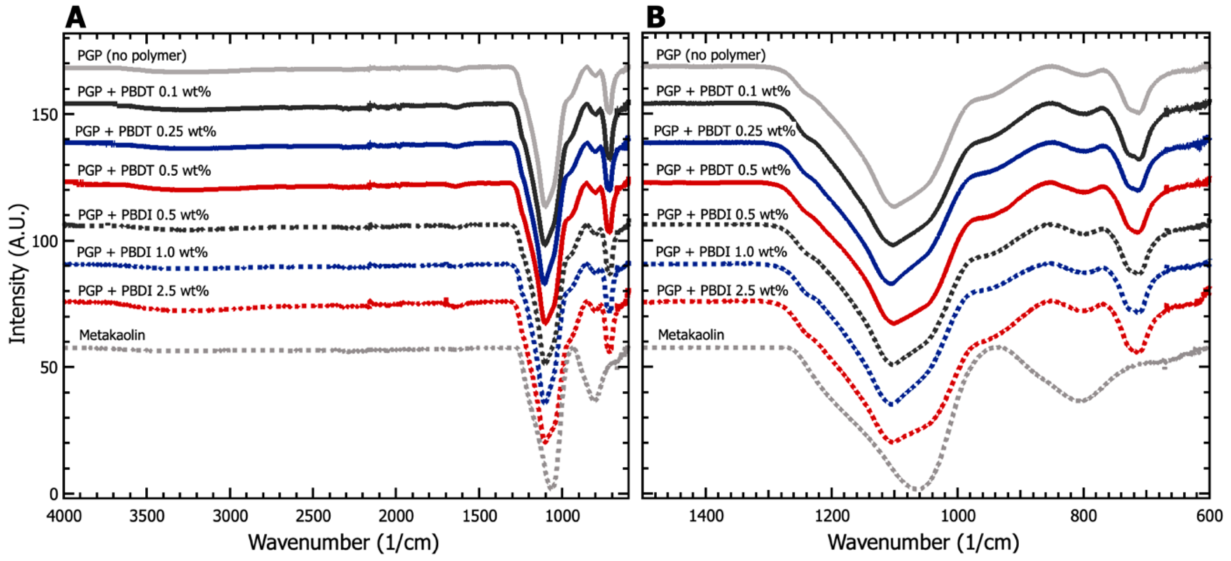


**Figure S4**. Fourier transform infrared radiation spectroscopy (FTIR) analysis of PGP and PGP + Polymer composites. (**A**) Spectrum from 4000–600 $cm^{-1}$. (**B**) Spectrum from 1500-600 $cm^{-1}$.

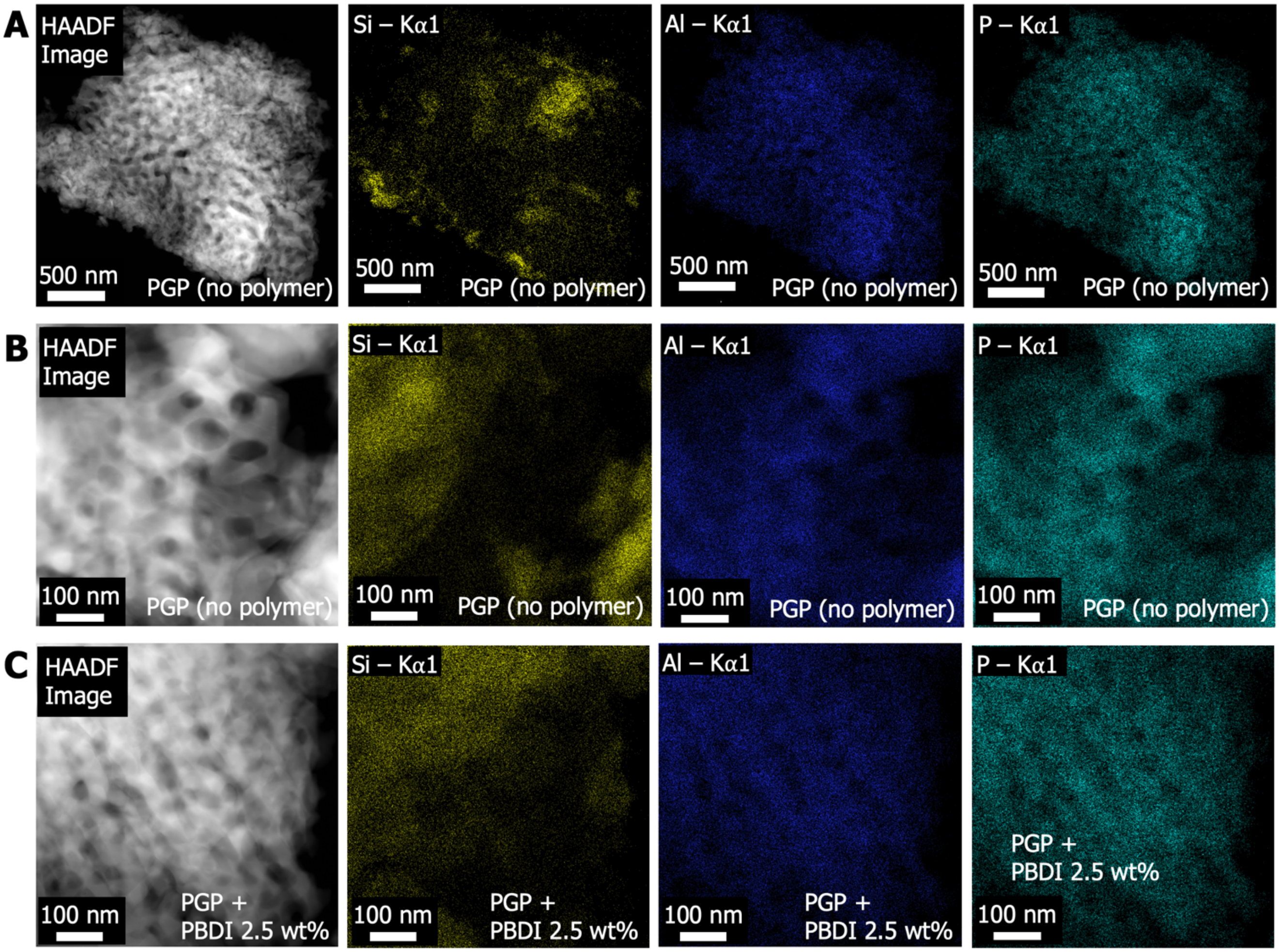


**Figure S5**. STEM high angle annular dark field (HAADF) and colored EDS Kα1 emission maps of Si (yellow), Al (blue), P (cyan). (**A**) PGP without polymer at 500 nm scale, (**B**) PGP without polymer at 100 nm scale, and (**C**) PGP + PBDI 2.5 wt. % at 100 nm scale.

**Table S2**. EDS chemical composition of PGP+Polymer plaques in atomic %.

| Plaque | Si-Rich | | | | Al/P-Rich | | | |
|---|---|---|---|---|---|---|---|---|
| | O At. % | Si At. % | Al At. % | P At. % | O At. % | Si At. % | Al At. % | P At. % |
| PGP (no polymer) | 63.67 | 16.02 | 9.23 | 8.23 | 63.62 | 8.18 | 13.37 | 13.51 |
| PGP (no polymer) | 66.54 | 18.93 | 9.12 | 7.40 | 65.66 | 4.17 | 15.38 | 14.78 |
| PGP (no polymer) | 65.44 | 21.01 | 5.98 | 7.04 | 66.32 | 3.99 | 15.07 | 14.02 |
| PGP+PBDT 0.1 wt. % | 65.16 | 17.93 | 9.26 | 7.23 | 66.26 | 4.73 | 14.32 | 14.40 |
| PGP+PBDT 0.1 wt. % | 66.36 | 17.58 | 9.32 | 8.52 | 66.02 | 7.97 | 15.41 | 14.31 |
| PGP+PBDT 0.1 wt. % | 65.78 | 22.79 | 7.42 | 8.12 | 64.31 | 5.63 | 14.82 | 14.02 |
| PGP+PBDT 0.25 wt. % | 63.02 | 20.77 | 6.45 | 7.02 | 64.95 | 7.12 | 15.42 | 14.53 |
| PGP+PBDT 0.25 wt. % | 67.53 | 22.05 | 7.84 | 6.24 | 67.91 | 4.35 | 15.48 | 14.24 |
| PGP+PBDT 0.25 wt. % | 66.72 | 18.63 | 6.99 | 6.57 | 65.37 | 6.63 | 16.03 | 13.94 |
| PGP+PBDT 0.5 wt. % | 65.87 | 21.72 | 5.21 | 5.28 | 65.20 | 7.92 | 14.95 | 13.99 |
| PGP+PBDT 0.5 wt. % | 64.82 | 17.92 | 9.92 | 5.42 | 63.06 | 3.46 | 15.41 | 13.92 |
| PGP+PBDT 0.5 wt. % | 64.46 | 21.86 | 7.43 | 6.94 | 66.84 | 7.03 | 15.25 | 14.07 |
| PGP+PBDI 0.5 wt. % | 65.09 | 17.35 | 6.02 | 6.97 | 65.24 | 5.34 | 15.12 | 14.53 |
| PGP+PBDI 0.5 wt. % | 65.57 | 22.39 | 5.93 | 7.18 | 64.12 | 5.49 | 15.08 | 14.99 |
| PGP+PBDI 0.5 wt. % | 66.82 | 18.47 | 8.32 | 8.32 | 66.72 | 6.81 | 15.01 | 14.70 |
| PGP+PBDI 1.0 wt. % | 66.79 | 15.97 | 9.63 | 8.11 | 65.96 | 3.82 | 15.92 | 14.52 |
| PGP+PBDI 1.0 wt. % | 68.54 | 17.64 | 7.94 | 8.02 | 67.42 | 5.62 | 16.35 | 14.02 |
| PGP+PBDI 1.0 wt. % | 63.83 | 21.63 | 5.07 | 7.32 | 63.54 | 4.99 | 14.83 | 14.56 |
| PGP+PBDI 2.5 wt. % | 66.01 | 21.89 | 5.99 | 7.26 | 63.21 | 7.44 | 14.42 | 13.97 |
| PGP+PBDI 2.5 wt. % | 64.87 | 20.03 | 7.42 | 7.03 | 62.99 | 4.23 | 15.14 | 14.34 |
| PGP+PBDI 2.5 wt. % | 62.92 | 22.53 | 5.92 | 6.83 | 67.41 | 6.51 | 15.02 | 14.41 |

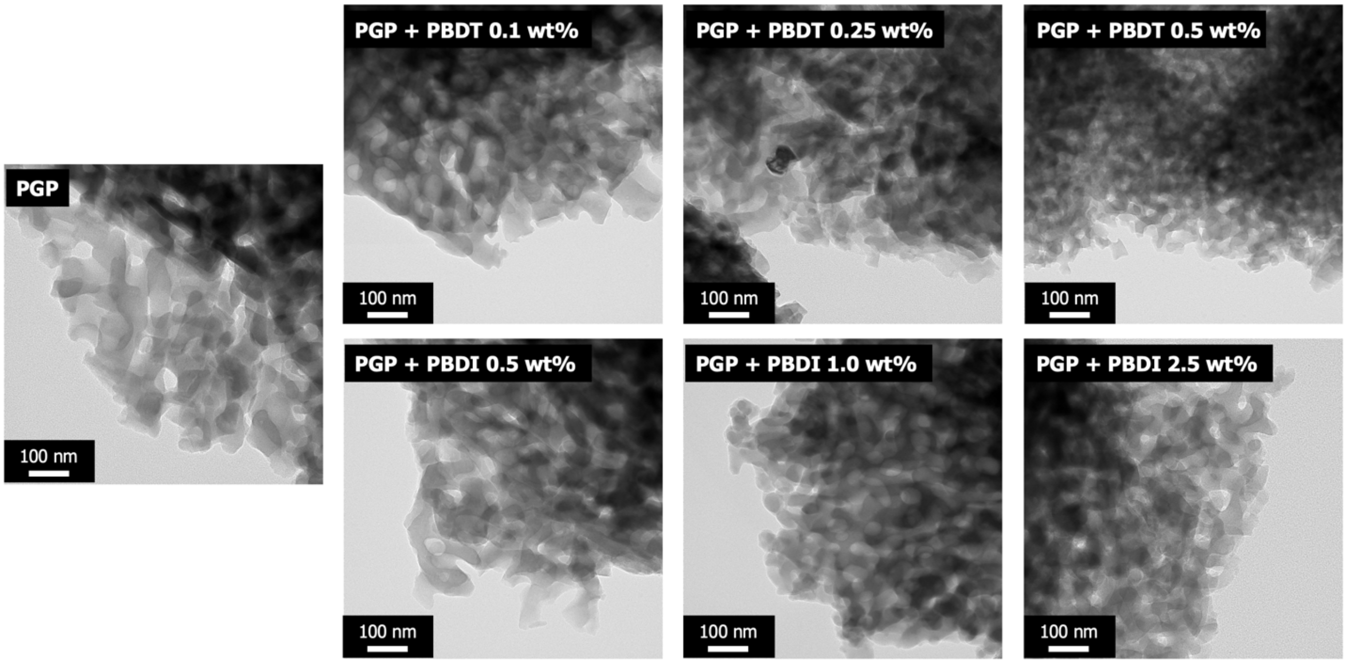


**Figure S6**. TEM images of (**left**) PGP, (**top**) PGP + PBDT, and (**bottom**) PGP + PBDI composites.

## References

1. Wang, W.K.; Chen, J.J.; Zhang, X.; Huang, Y.X.; Li, W.W.; Yu, H.Q. Self-Induced Synthesis of Phase-Junction TiO2 with a Tailored Rutile to Anatase Ratio below Phase Transition Temperature. *Sci. Rep.* **2016**, *6*, 20491, doi:10.1038/srep20491.
2. Uddin, M.J.; Cesano, F.; Chowdhury, A.R.; Trad, T.; Cravanzola, S.; Martra, G.; Mino, L.; Zecchina, A.; Scarano, D. Surface Structure and Phase Composition of TiO2 P25 Particles After Thermal Treatments and HF Etching. *Front. Mater.* **2020**, *7*, 192, doi:10.3389/fmats.2020.00192.
3. Debnath, R.; Chaudhuri, J. Surface-Bound Titania-Induced Selective Growth and Stabilization of Tridymite Aluminum Phosphate. *J. Solid State Chem.* **1992**, *97*, 163–168, doi:10.1016/0022-4596(92)90021-M.
4. Wagemaker, M.; Kearley, G.J.; Van Well, A.A.; Mutka, H.; Mulder, F.M. Multiple Li Positions inside Oxygen Octahedra in Lithiated TiO2 Anatase. *J. Am. Chem. Soc.* **2003**, *125*, 840–848, doi:10.1021/ja028165q.
5. Neder, R.B.; Burghammer, M.; Grasl, T.; Schulz, H.; Bram, A.; Fiedler, S. Refinement of the KaolinitesStructure from Single-Crystal Synchrotron Data. *Clays Clay Miner.* **1999**, *47*, 487–494, doi:https://doi.org/10.1346/CCMN.1999.0470411.
